# A brittle star-like robot capable of immediately adapting to unexpected physical damage

**DOI:** 10.1098/rsos.171200

**Published:** 2017-12-13

**Authors:** Takeshi Kano, Eiki Sato, Tatsuya Ono, Hitoshi Aonuma, Yoshiya Matsuzaka, Akio Ishiguro

**Affiliations:** 1 Research Institute of Electrical Communication, Tohoku University, 2-1-1 Katahira, Aoba-Ward, Sendai 980-8577, Japan; 2 Research Center of Mathematics for Social Creativity, Research Institute for Electronic Science, Hokkaido University, N12W7, Kita-Ward, Sapporo, Hokkaido 060-0812, Japan; 3 Japan Science and Technology Agency CREST, 4-1-8 Honcho, Kawaguchi, Saitama 332-0012, Japan; 4 Department of Physiology, Graduate School of Medicine, Tohoku University, 2-1 Seiryo-machi, Aoba-Ward, Sendai 980-8575, Japan

**Keywords:** decentralized control, resilient robot, brittle star

## Abstract

A major challenge in robotic design is enabling robots to immediately adapt to unexpected physical damage. However, conventional robots require considerable time (more than several tens of seconds) for adaptation because the process entails high computational costs. To overcome this problem, we focus on a brittle star—a primitive creature with expendable body parts. Brittle stars, most of which have five flexible arms, occasionally lose some of them and promptly coordinate the remaining arms to escape from predators. We adopted a synthetic approach to elucidate the essential mechanism underlying this resilient locomotion. Specifically, based on behavioural experiments involving brittle stars whose arms were amputated in various ways, we inferred the decentralized control mechanism that self-coordinates the arm motions by constructing a simple mathematical model. We implemented this mechanism in a brittle star-like robot and demonstrated that it adapts to unexpected physical damage within a few seconds by automatically coordinating its undamaged arms similar to brittle stars. Through the above-mentioned process, we found that physical interaction between arms plays an essential role for the resilient inter-arm coordination of brittle stars. This finding will help develop resilient robots that can work in inhospitable environments. Further, it provides insights into the essential mechanism of resilient coordinated motions characteristic of animal locomotion.

## 1. Introduction

Robots are now required to work in harsh environments inaccessible to humans such as disaster areas [1,2], distant planets [3] and deep oceans [4]. A major obstacle in this regard is that robots cannot cope with physical damage to their bodies. One possible solution is to preprogram contingency plans for anticipated failure modes [5–8], yet this approach fails under unexpected events. Engineers have attempted to overcome this problem using several techniques such as learning [9–15] and trial-and-error methods [16]. However, these methods [9–16] require considerable amounts of time (several tens of seconds to several minutes) to respond to unexpected physical damage while incurring high computational costs. In contrast, living organisms survive in harsh environments by coping with unexpected physical damage in real time. The key insight here is that even primitive creatures from amoeba to namatode exhibit such adaptability by coordinating their body parts appropriately [17,18]. This fact indicates that real-time response to unexpected physical damage can be achieved with small amount of computational costs, unlike the control schemes used in conventional robots [9–16]. Therefore, clarifying the core mechanism for resilient coordinated motion in living organisms could pave the way for developing robots that can immediately cope with unexpected physical damage.

**Figure 1.**
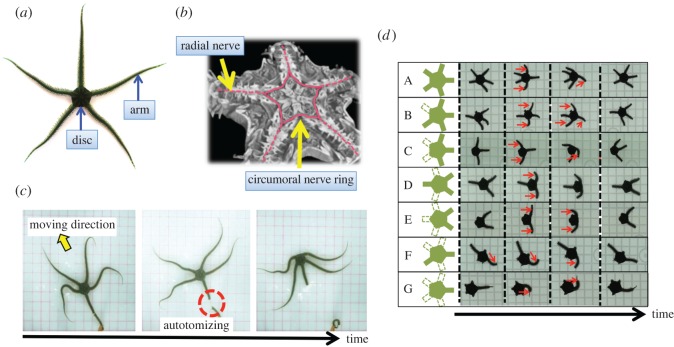
Body structure and motion of a brittle star. (*a*) Overview of a brittle star (*Ophiarachna incrassata*). Five flexible arms radiate from a central disc. The body of an intact brittle star is radially symmetrical. (*b*) Micro-computed tomography image of a brittle star. The nervous system is indicated by pink lines. Radial nerves that innervate the arms are connected via a circumoral nerve ring located in the central disc. The method for obtaining the images is provided in appendix A. (*c*) Photographs of a brittle star autotomizing one of its arms when hypertonic seawater is applied while the tip of the arm is immobilized. (*d*) Locomotion of brittle stars whose arms have been trimmed or amputated. Seven types of morphology (A–G) were examined. The direction of motion was from left to right. Photographs were taken around every 0.5–1.0 s. The arrows denote the arms that mainly contributed to the locomotion.

In this paper, we focus on a brittle star, a phylum of echinoderm that locomote on the sea floor by coordinated rhythmic movements of their five multi-segmented arms (figure 1*a*) [19–28]. Two notable features of this species motivated us to study its locomotion as a biological model of a resilient robot. First, a brittle star lacks a central nervous system but instead possesses a rather simple distributed nervous system consisting of radial nerves along its arms, which join a circumoral nerve ring (figure 1*b*; electronic supplementary material, video S1, 1 : 13–1 : 31) [27]. Despite the lack of a sophisticated centralized controller, it assigns distinct roles to individual arms and coordinates their movements to propel the body [21–26]. Second, this species has outstanding resilience to bodily damage. Even after arbitrary loss of its arms, it promptly reassigns the roles to and reorganizes the coordination of the remaining arms to resume locomotion (figure 1*c*; electronic supplementary material, video S1, 0 : 55–1 : 13) [22]. These features made it a suitable model that overcomes the limitations of previously proposed techniques [5–16].

In this study, we adopted a synthetic approach [29,30] to deduce the decentralized control mechanism that underlies the resilience of brittle stars' locomotion. Specifically, we inferred the decentralized control mechanism that underlies brittle star locomotion by constructing a simple model from a macroscopic viewpoint, based on anatomical and behavioural findings of this creature. The inferred mechanism was implemented in a brittle star-like robot to demonstrate that it can immediately adapt to physical damage by coordinating its arms in a manner similar to a living brittle star. We adopted this approach because it is difficult to physiologically examine the functions of neuromuscular systems of behaving brittle stars, and even if it is possible, the complex behaviour of the organism can hardly be reconstructed from the functions of its individual neurons and muscles. Our approach has the advantage that it enables us to capture the possible essential mechanism, or the minimum requirement of the brittle stars' resilient locomotion.

## 2. Related works on brittle stars

### 2.1. Anatomical studies

Brittle stars have a circular body disc and typically five radiating arms (figure 1*a*). Each arm consists of a series of segments, each containing a roughly discoidal vertebral ossicle. Adjacent ossicles are linked by four muscle blocks, which enables the arm to bend both in the horizontal and vertical directions [28]. The arm movements are innervated by a simple distributed nervous system. Specifically, brittle stars have a ‘circumoral nerve ring’ that surrounds the disc and connects to ‘radial nerves’ running along the arms (figure 1*b*; electronic supplementary material, video S1, 1 : 13–1 : 31) [27]. Each arm communicates with its two adjacent arms via the nerve ring [22].

### 2.2. Behavioural studies

Brittle stars have a locomotion strategy distinguished from any other metazoan: arms with highly muscularized internal skeletons coordinate powerful strides for rapid movement across the ocean floor [23]. Despite the lack of a sophisticated centralized controller, brittle stars assign distinct roles to individual arms and coordinate their movements to propel the body [21–26]. When a stimulus is encountered and locomotion becomes necessary, each arm is assigned one of three roles in the gait corresponding with its position relative to the requisite direction of movement [21,22,25,26]. One arm is designated as the centre limb, two as the forelimbs and two as hindlimbs. The centre limb is the arm parallel to the direction of movement. The forelimbs are the primary structures that work in coordination to move the organism forward, and the hindlimbs take a minimal role in propulsion. When the centre limb is anterior to the direction of desired disc movement (during the locomotor mode, referred to as ‘rowing’), the left and right forelimbs are adjacent to the centre limb, and the remaining two take the role as the hindlimbs. When the centre limb is posterior to the direction of movement (referred to as ‘reverse rowing’), the forelimbs are the most anterior limbs, while the hindlimbs flank the centre limb [21,23]. Each arm is capable of assuming any of the three roles. Therefore, to change direction, the animal simply reassigns the roles of the arms [23]. This system allows these organisms to move in every direction equally without rotating the body to turn, as would need to occur in a bilateral organism.

Further, brittle stars can seamlessly modify their locomotion strategy to accommodate a lost or inoperative arm [19,20,22,31]. For example, a brittle star can autotomize some of its arms and coordinate the remaining arms to evade predators or harmful stimuli (figure 1*c*; electronic supplementary material, video S1, 0 : 55–1 : 13) [19,20]. Brittle stars whose arms are amputated surgically in various ways can also maintain locomotion by coordinating the remaining arms [22].

### 2.3. Mathematical and robotic studies

Brittle star locomotion has also attracted attention in the fields of mathematics and robotics. For example, Lal *et al*. [32] developed a brittle star-like modular robot. They let the robots learn their movements by using a genetic algorithm so as to coordinate each other and generate locomotion. However, as the ‘performance phase’ of the robot is completely separated from the ‘learning phase’ that requires a certain amount of time, the robot cannot behave adaptively in real time.

In contrast, we have proposed a decentralized control mechanism for the locomotion of brittle stars with five arms, based on a synthetic approach [25,26]. Spontaneous role assignment of rhythmic and non-rhythmic arm movements was modelled by using an active rotator model that can describe both oscillatory and excitatory properties. The proposed mechanism was validated via simulations [25] and robot experiments [26].

## 3. Behavioural experiments

We first performed behavioural experiments wherein we analysed the resilience of the subjects' locomotion by amputating some of their arms from their bases (figure 1*d*). The non-amputated arms originally had a large number of degrees of freedom because each consisted of numerous ossicles and muscles [28,33]. In our experiments, however, we trimmed them to minimize their degrees of freedom so that we could focus on their inter-arm coordination mechanism.

We used adult subjects (*Ophiarachna incrassata*) raised in seawater with a density of 1.022 ± 0.002 g ml^−1^ and a temperature of 26.5 ± 0.5°C. We removed 10 subjects' arms surgically at 12 ± 4 mm and less than 2 mm from the proximal end for the trimmed and amputated arms, respectively, and observed the subjects' locomotion after the amputation. For each subject, several trials were performed under the same configurations. The number of trials for each configuration is shown in table 1. It is noted that slight length variations of the trimmed arms did not affect locomotion significantly. Further, although some arms regenerated after they had been cut, the regenerated portions were not sufficiently large to contribute to locomotion.

**Table 1.** Number of trials for each subject and the robot. Symbols A–G correspond to those in figure 1*d* and figure 8*b*.

subject	A	B	C	D	E	F	G
no. 1	5	4	—	8	—	11	6
no. 2	7	4	4	—	8	—	12
no. 3	5	15	13	—	—	—	—
no. 4	6	7	—	—	—	—	—
no. 5	—	39	11	—	—	6	5
no. 6	18	15	—	—	—	—	—
no. 7	12	28	—	9	—	—	—
no. 8	20	11	—	14	8	—	—
no. 9	—	—	—	—	9	—	—
no. 10	—	—	—	—	—	8	—
robot	11	10	11	10	10	10	11

**Figure 2.**
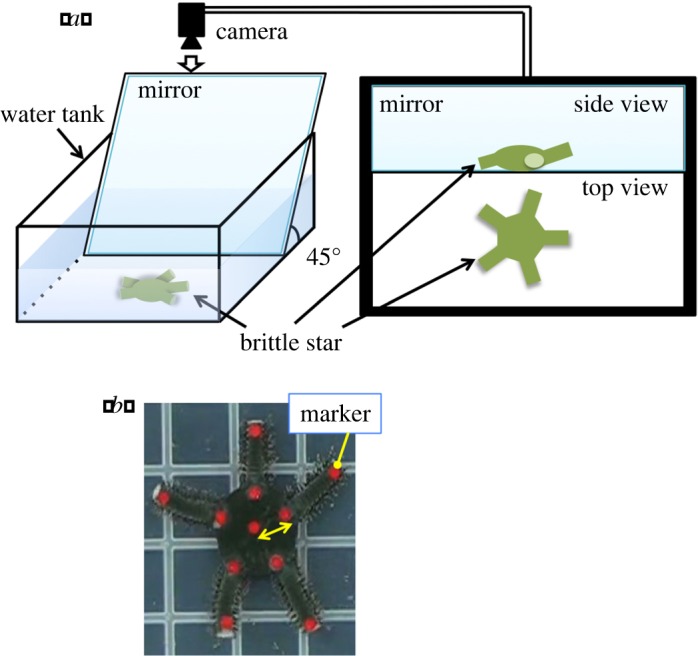
Behavioural experiments. (*a*) Experimental set-up. The motion of the subjects was monitored using an overhead camera. A mirror was placed beside the subjects to simultaneously monitor their top and side views. (*b*) Markers on a real brittle star, which were used to calculate the indices.

The experimental set-up is shown in figure 2*a*. A square plastic container was filled with seawater and the subjects were placed at the bottom. A mirror was placed beside the subjects at an inclination of around *π*/4. Top and side views of the subjects were monitored simultaneously using an overhead camera.

During the experiments, several subjects became inactive after the surgery and only sporadically exhibited voluntary movements. Therefore, we stimulated the discs of the subjects using hypertonic seawater in order to initiate their motion. The subjects tended to move in the direction opposite to the stimulated points. We used the data obtained from trials in which the subjects moved in specific directions (the directions shown in figure 1*d*).

For the experiments, we attached markers to the subjects' bodies using adhesive (figure 2*b*) to facilitate motion capture tracking. The data obtained were used to calculate the locomotion velocity and inter-arm coordination index described below. The analysis was performed from the commencement of motion until the end of the motion or until the subjects reached the side walls of the container. However, data obtained in the following cases were omitted: (i) when the evaluation time was less than 10 s or (ii) when the direction of motion changed during the experiment.

**Figure 3.**
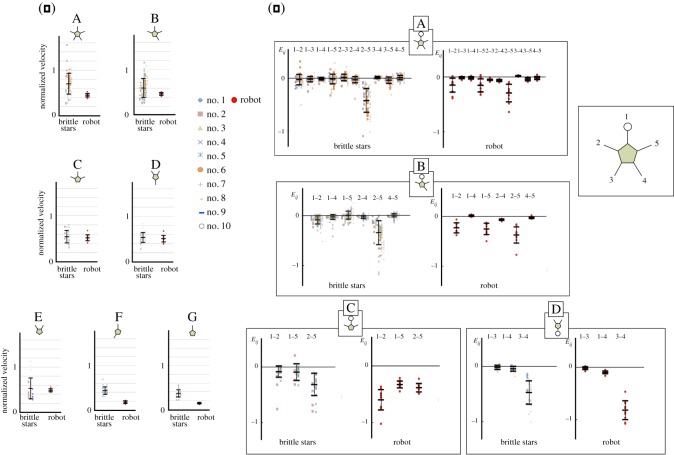
Quantitative comparison of the brittle star and the robot. (*a*) Normalized locomotion velocity. (*b*) Inter-arm coordination index *E_ij_*. The morphology examined is shown at the top of each graph. The numbers at the top of each graph denote the arm pairs, and the arm numbers are shown in the inset. The open circles in the schematics denote arm 1. The bars in the graphs denote the mean and variance.

Photographs and a video of the subjects' locomotion are shown in figure 1*d* and electronic supplementary material, video S1, 1 : 52–2 : 11 and 5 : 27–5 : 38, respectively. When the five trimmed arms remained, one arm is designated as the centre limb that orients the direction of movement, two as the forelimbs that move synchronously to propel the body, and two as hindlimbs that take a minimal role in propulsion, in a similar manner as intact brittle stars (figure 1*d*(A)). When one leg was removed, the subjects were able to execute rowing without loss in capabilities by assigning the missing limb in the ‘hindlimb’ position (figure 1*d*(B)). Similarly, when two adjacent arms were removed, the subjects continued to row without decrease in speed or ability by assigning the missing limbs as ‘hindlimbs’; they continued to use the remaining arms as the centre and forelimbs (figure 1*d*(C)). However, when the configuration changed and the two arms adjacent to a remaining arm were removed, the subjects reverted to ‘reverse rowing’ using the two remaining neighbouring arms as forelimbs, with the third trailing posteriorly (figure 1*d*(D)). The strategy persisted after the trailing third arm was removed (figure 1*d*(E)). When three arms were removed so that no adjacent arms remained, the arm orienting the direction of movement swung to both left and right to pull the body forward, while the other remaining arm took a minimal role in propulsion (figure 1*d*(F)). When only one arm remained, the subjects were still able to move forward by swinging the arm to both the left and right, pulling the body forward (figure 1*d*(G)). The locomotion velocity of the subjects with only one trimmed arm (configuration G) was more than 50% of that with five trimmed arms (configuration A) (figure 3*a*). We also observed the locomotion of brittle stars having one or several long flexible arms, and found that they move in a similar manner as figure 1*d*, although the motion was somewhat complex (electronic supplementary material, video S2).

To evaluate the coordinated arm motion of the trimmed-arm subjects quantitatively, we defined the inter-arm coordination index *E_ij_* as

3.1 $$E_{ij}=\frac{1}{T}\sum_{t=1}^{T}\omega_{i}(t)\omega_{j}(t),$$

where *i* and *j* are the arm indices (i,j=1,2,3,4,5), $\omega_{i}(t)$ is the angular velocity of the *i*th arm at time step *t* and *T* is the total number of time steps during the observation. Further, *E_ij_* is positive or negative when the *i*th and *j*th arms move synchronously in the same or opposite directions, respectively, and it is close to zero when they are inactive or their motions are uncorrelated. The *E_ij_* values for the subjects with more than two arms are shown in figure 3*b*. It is evident that |*E_ij_*| for one of the combinations of *i* and *j* is larger than that for the other combinations in each configuration. The arm combination for which the |*E_ij_*| value is large varies when some of the arms are amputated: |*E_ij_*| for the distant arm pair (|*E*_25_|) is large for configurations A–C while |*E_ij_*| for the adjacent arm pair (|*E*_34_|) is large for configuration D. This result suggests that the coordination pattern is not predetermined but changes depending on the configuration of the body in order to adapt to the amputation.

**Figure 4.**
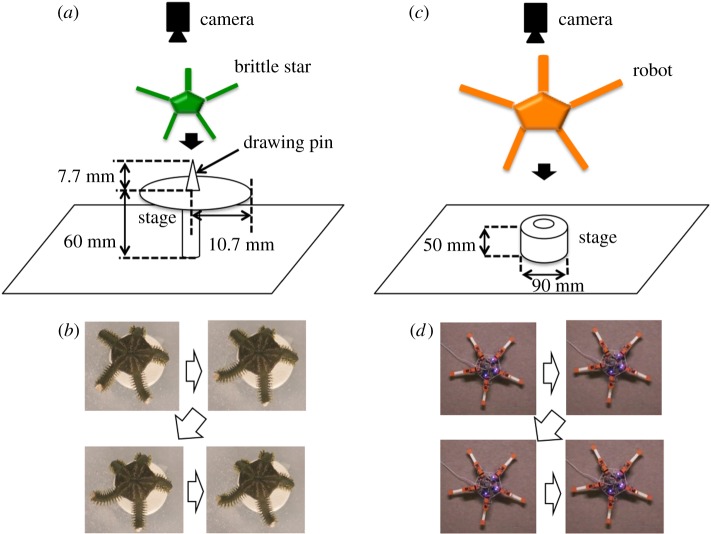
Experiments without ground contact of the arms. (*a*) Experimental set-up for the behavioural experiment. In seawater, the centre of the central disc of a brittle star was fixed on a stage to which a drawing pin was attached; thus, the arms did not touch the ground. (*b*) Photographs of a brittle star on the stage, which were taken around every 1.0 s. (*c*) Experimental set-up for the robot experiment. The robot was placed on a stage such that its arms did not touch the ground. (*d*) Photographs of the robot on the stage, which were taken around every 1.0 s.

The above-mentioned coordination pattern change is possibly triggered by changes in the interaction between the body and the environment due to the amputation. To test this possibility, we investigated how the deprivation of the ground contact of the arms affects the inter-arm coordination using living brittle stars. Specifically, the subjects were picked up after several seconds of locomotion on the ground and fixed on a stage with a drawing pin so that their arms did not touch the ground (figure 4*a*). After observing their arm motion for several seconds, the subjects were placed on the ground again. It was found that the subject lost its arm coordination when it was fixed on the stage (figure 4*b*), while it immediately recovered its arm coordination when we placed it on the ground again (electronic supplementary material, video S3). We confirmed that this tendency was observed for three subjects, each of which underwent five trials. From this result, we found that the interaction between the arms and the ground plays a crucial role in inter-arm coordination.

## 4. Mathematical modelling

In this section, a decentralized control mechanism for the inter-arm coordination is inferred from the above-mentioned behavioural findings, based on which a mathematical model is proposed. Although a brittle star likely determines its direction of motion by integrating sensory information detected at each part of its body, we ignore this process and assume that the direction of motion is predetermined in our model. This assumption is employed because our focus in this study is the inter-arm coordination mechanism after the direction of motion is determined. Moreover, it is assumed in our model that governing equations for controllers ((4.3)–(4.8) described below) do not change under any physical damage.

**Figure 5.**
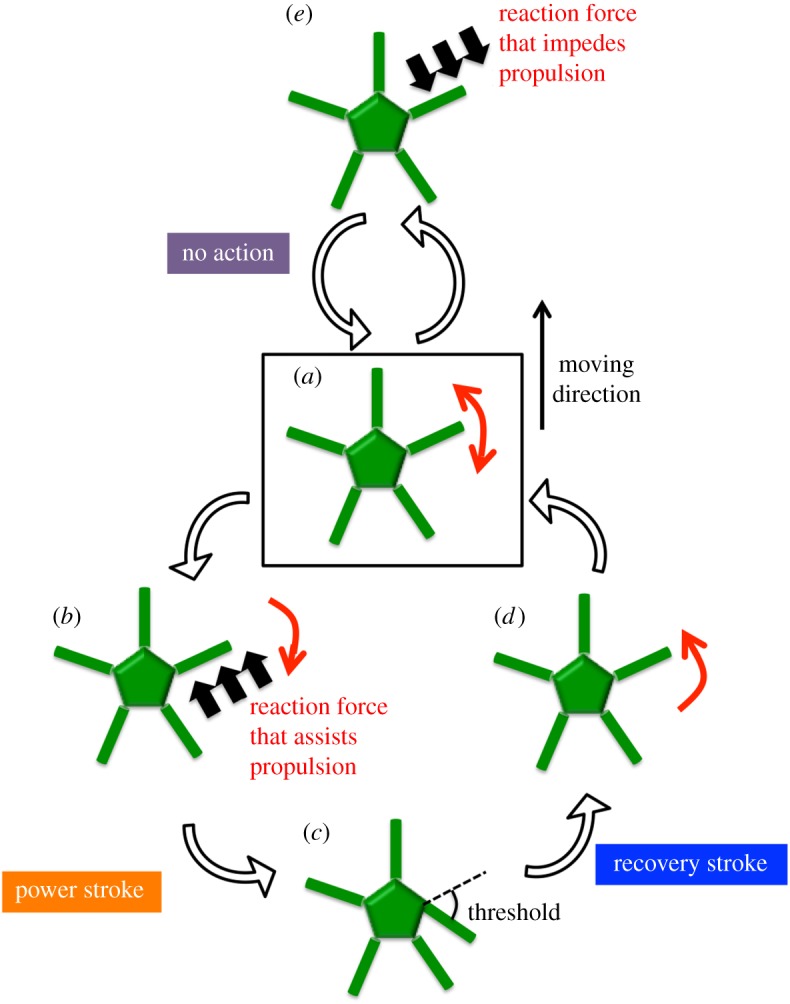
Outline of the decentralized control mechanism inferred from the behavioural experiments. (*a*) Each arm moves randomly and obtains a response from the environment. (*b*) If the reaction force assists propulsion, the arm pushes against the ground. (*c*,*d*) The arm lifts off from the ground and moves forward when the joint angle at the proximal end reaches a certain threshold. (*e*) When the reaction force impedes propulsion, the arm does not push against the ground.

With the absence of a centralized control system and the arm–ground interaction being the critical factor for the inter-arm coordination, we inferred that the following mechanism underlies the resilience of the brittle stars' locomotion (figure 5 and electronic supplementary material, video S1, 2 : 12–3 : 01). First, each arm then moves randomly to detect the reaction force against the ground (figure 5*a*). If the reaction force assists with propulsion, the arm pushes against the ground, i.e. a power stroke begins (figure 5*b*). Then, a recovery stroke begins when the joint angle reaches a certain threshold (figure 5*c*,*d*). On the other hand, if the reaction force impedes propulsion, no action is generated (figure 5*e*). It should be noted that in this mechanism, the motion of each arm is determined by the reaction force from the environment, which should be physically affected by the motion of the other arms; hence, physical interaction is crucial for achieving inter-arm coordination.

**Figure 6.**
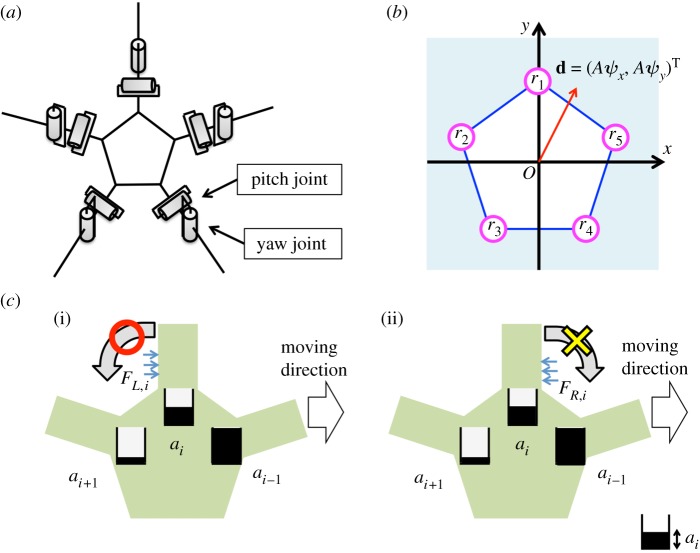
Schematics for the proposed model. (*a*) Schematic of the body system. (*b*) Definitions of the vectors **d** and **r***_i_*. (*c*) Evaluation of reaction force from the environment when $a_{i-1}>a_{i}>a_{i+1}$. The *i*th arm pushes against the ground when it obtains a reaction force from the left because $a_{i}>a_{i+1}$. On the other hand, the *i*th arm does not push against the ground when it obtains a reaction force from the right because $a_{i}<a_{i-1}$.

A mathematical model was proposed based on the above-mentioned mechanism. The schematic of the body system is shown in figure 6*a*. The body consists of a central disc and five arms. Because the trimmed-arm subjects had only a few degrees of freedom in each arm, each arm has only two degrees of freedom, i.e. yaw and pitch joints. It was assumed that each arm can detect the ground reaction force parallel to the ground.

Based on the predetermined direction of motion, which is denoted by vector **d**, the parameters *a_j_* (*j* = 1, 2, 3, 4, 5) were defined for each arm as follows. The vectors **r***_j_*, which consist of the vertices of a regular pentagon in the *xy* plane (figure 6*b*), are defined as

4.1 $${\text{r}}_{j}={\left[-\sin\left\{\frac{2}{5}\pi (\;j-1)\right\},\cos\left\{\frac{2}{5}\pi (\;j-1)\right\}\right]}^{T},$$

and *a_j_* is given by

4.2 $$a_{j}={\text{r}}_{j}\cdot \text{d}\text{.}$$

Thus, *a_j_* is large when the proximal end of the *j*th arm is oriented towards the direction of motion from the viewpoint of the centre of the central disc.

The joint torque is determined according to proportional–derivative control, and the time evolution of the target joint angles of the *i*th arm is defined as follows:

4.3 $${\dot{\Phi }}_{i}=c(\Xi_{i}+{\text{P}}_{i}+{\text{R}}_{i}-\Phi_{i}),$$

where *c* is a positive constant and $\Phi_{i}={\left[\phi_{\mathrm{yaw},i},\phi_{\mathrm{pitch},i}\right]}^{T}$ with $\phi_{\mathrm{yaw},i}$ and $\phi_{\mathrm{pitch},i}$ being the target angles for the yaw and pitch joints, respectively. Note that the signs of the yaw and pitch joint angles are taken as positive when the arm bends leftward and upward with respect to the central disc, respectively. The vectors Ξ*_i_*, **P***_i_* and **R***_i_* contribute to the generation of the noise, power stroke and recovery stroke, respectively. Hereafter, we describe mathematical formulae for these terms.

The noise vector Ξ*_i_* is defined as

4.4 $$\Xi_{i}=\left[\begin{array}{c} \alpha_{\mathrm{yaw}}\xi_{\mathrm{yaw},i}\\ \alpha_{\mathrm{pitch}}\xi_{\mathrm{pitch},i} \end{array}\right],$$

where *α*_yaw_ and *α*_pitch_ are positive constants and *ξ*_yaw,*i*_ and *ξ*_pitch,*i*_ are uniform random numbers in the range [−1,1], which vary with a fixed interval. It should be noted that the noise is essential because the robot cannot interact with the environment in the absence of noise.

The power stroke vector **P***_i_* is defined as

4.5 $${\text{P}}_{i}=\left[\begin{array}{c} -\beta_{\mathrm{yaw}}(U_{R,i}-U_{L,i})\\ -\beta_{\mathrm{pitch}}(U_{R,i}-U_{L,i} \end{array}\right],$$

where *β*_yaw_ and *β*_pitch_ are positive constants, and

4.6 $$\begin{array}{rl} U_{R,i} & =\max[a_{i}-a_{i-1},0]F_{R,i}\\ \mathrm{and}\;U_{L,i} & =\max[a_{i}-a_{i+1},0]F_{L,i}. \end{array}\}$$

Here, *F_R_*_,*i*_ and *F_L_*_,*i*_ denote the reaction forces acting on the right- and left-hand sides of the *i*th arm, respectively. In (4.6), the terms max[*a_i_* − *a_i_*_−1_, 0] and max[*a_i_* − *a_i_*_+1_, 0] were introduced to evaluate whether the reaction force from the environment assists propulsion. For example, let us consider the case in which the (*i* − 1)th arm is oriented toward the moving direction, i.e. *a_i_*_−1_ > *a_i_* > *a_i_*_+1_ (figure 6*c*). When the *i*th arm experiences a reaction force from the left, i.e. a reaction force assisting propulsion (figure 6*c*(i)), *U_L_*_,*i*_ increases according to (4.6). Then, from (4.3) and (4.5), the target yaw and pitch joint angles of the *i*th arm increase and decrease, respectively, causing the *i*th arm to push against the ground and propelling the body in the direction of motion. On the other hand, when the *i*th arm receives a reaction force from the right, i.e. a reaction force that hinders propulsion (figure 6*c*(ii)), *U_R_*_,*i*_ does not increase because max[*a_i_* − *a_i_*_−1_, 0] is zero. Thus, the target joint angles of the *i*th arm do not change and no action is generated.

The recovery stroke vector **R***_i_* is defined as

4.7 $${\text{R}}_{i}=\left[\begin{array}{c} \gamma_{\mathrm{yaw}}(S_{R,i}-S_{L,i})\\ \gamma_{\mathrm{pitch}}(S_{R,i}-S_{L,i}) \end{array}\right],$$

where *γ*_yaw_ and *γ*_pitch_ are positive constants, and *S_R_*_,*i*_ and *S_L_*_,*i*_ are given by

4.8 $$\begin{array}{rl} S_{R,i} & =\frac{1}{2}[1+\tanh\{\kappa_{s}(-\theta_{\mathrm{yaw},i}-\theta_{th})\}]\\ \mathrm{and}\;S_{L,i} & =\frac{1}{2}[1+\tanh\{\kappa_{s}(\theta_{\mathrm{yaw},i}-\theta_{th})\}], \end{array}\}$$

where *κ_s_* is a positive constant, and *θ*_yaw,*i*_ is the yaw joint angle of the *i*th arm. When the joint angle reaches the threshold angle *θ_th_* or −*θ_th_*, *S_L,i_* or *S_R_*_,*i*_ increases, respectively; thus, the target joint angle changes, causing the *i*th arm to move in the opposite direction by lifting off from the ground.

In sum, the noise vector Ξ*_i_* is essential for detecting the reaction force against the ground (figure 5*a*). The power stroke vector **P***_i_* is **0** unless the *i*th arm detects a reaction force that assists propulsion; however, **P***_i_* acts in such a way that the *i*th arm can push against the ground when it detects an assistive reaction force (figure 5*b*). The recovery stroke vector **R***_i_* is nearly equal to **0** unless the yaw joint angle of the *i*th arm exceeds the threshold ±*θ_th_*; however, **R***_i_* acts in such a way that the *i*th arm moves itself forward when the yaw joint angle reaches the threshold (figure 5*c*,*d*).

It should be noted that the terms max[*a_i_* − *a_i_*_−1_, 0] and max[*a_i_* − *a_i_*_+1_, 0] in (4.6) could be based on neural—but not physical—interaction between adjacent arms. However, except for this neural interaction, no other neural interaction is assumed in our model, and physical interaction plays a crucial role in the arm coordination.

## 5. Robot experiment

**Figure 7.**
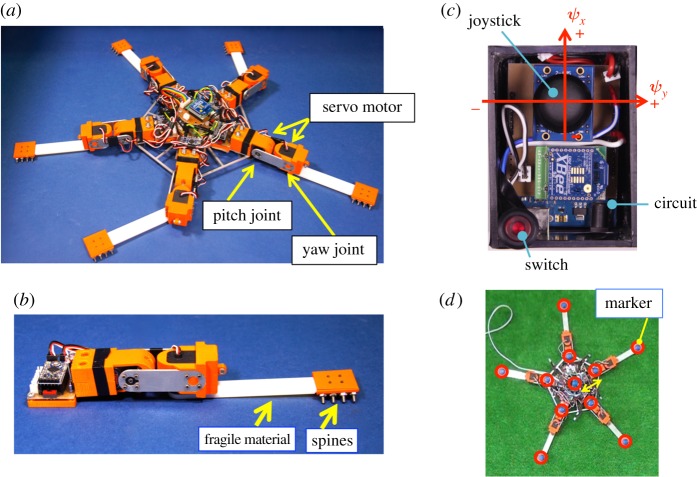
Structure of the robot. (*a*) Overview of the brittle star-like robot PENTABOT II. The robot consists of a central disc and five arms, each of which has yaw and pitch joints. (*b*) Detailed structure of each robot arm. Two servomotors are implemented to drive the joints. A fragile material was used for the distal part in order to allow examination of adaptability to damage. (*c*) Internal structure of remote controller. (*d*) Markers on the robot, which were used to calculate the indices.

To test the validity of the proposed decentralized control mechanism, we developed a robot that mimicked the trimmed-arm subject used in our behavioural experiments. Our robot consisted of a central disc with five arms (figure 7*a*). The diameter of the central disc, the arm length and the total weight were 0.18 m, 0.18 m and 1.06 kg, respectively. The power supply and circuit boards for communication between the microcomputers (Arduino Pro Mini; 5.0 V, 16 MHz) were embedded in the centre of the disc. Each arm of the robot had two degrees of freedom, i.e. yaw and pitch joints. A servomotor (RS-303MR) and a circuit board for controlling it were attached to each joint. The joint angles were detected by potentiometers implemented in the servomotors. The distal parts of the arms could be easily replaced between experiments and we used fragile materials, which were made using a 3D printer (Marubeni, Objet260 Connex2), for these parts when we investigated the responses to physical damage to the arms (figure 7*b*). Several spines were attached at the tip of each arm to ensure friction between the robot and the ground (figure 7*b*).

Reaction forces between the environment and each arm could not be measured directly with the developed robot. Instead, they were roughly estimated by measuring the deviation of the yaw joint angle from its target value. More specifically, the reaction forces acting on the right- and left-hand sides of the *i*th arm, *F_R_*_,*i*_ and *F_L_*_,*i*_, respectively, were estimated as

5.1 $$\begin{array}{rl} F_{R,i} & =\max[-\tanh\{\kappa_{f}(\phi_{\mathrm{yaw},i}-\theta_{\mathrm{yaw},i})\},0]\\ \mathrm{and}\;F_{L,i} & =\max[\tanh\{\kappa_{f}(\phi_{\mathrm{yaw},i}-\theta_{\mathrm{yaw},i})\},0], \end{array}\}$$

where *κ_j_* is a positive constant. In fact, *F_R_*_,*i*_ and *F_L_*_,*i*_ increase as the yaw joint angle deviates from its target value owing to the reaction forces acting on the right- and left-hand sides of the *i*th arm, respectively. The hyperbolic tangent functions in (5.1) were introduced because pressure sensory information is expected to saturate in real brittle stars as the reaction force increases.

The direction of motion of the robot was controlled via motor commands from a remote controller. A joystick was incorporated into the remote controller, and its displacements in the horizontal and vertical directions, *ψ_x_* and *ψ_y_*, respectively, were measured by two potentiometers (figure 7*c*). The values of *ψ_x_* and *ψ_y_* were wirelessly sent to the circuit in the disc of the robot. The vector that determines the direction of motion of the robot, **d**, is given by **d **= (*Aψ_x_*, *Aψ_y_*)^T^, where *A* is a positive constant.

**Figure 8.**
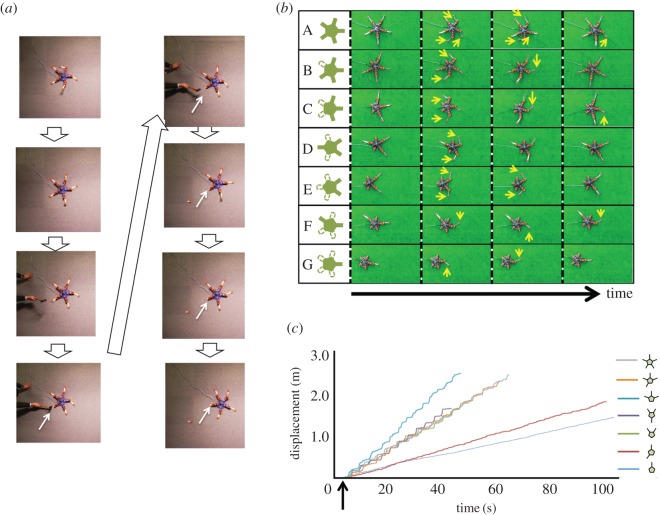
Results for the robot experiments. (*a*) Photographs of the distal part of one of the arms being destroyed during locomotion. The robot immediately adapted to the damage and maintained its locomotion. Arrows indicate damaged arms. The direction of motion was from left to right. Photographs were taken every 1.0 s. (*b*) Photographs of robot locomotion for which the direction of motion and configuration were nearly the same as those in the behavioural experiments shown in figure 1*d*. The direction of motion was from left to right. Photographs were taken around every 0.5–2.0 s. The arrows denote the main arms that contributed to locomotion. (*c*) Time evolution of the displacement of the robot. Data for the trials conducted in (*b*) are shown. The start point was set to the instant at which the robot was powered on. The arrow denotes the time at which the robot was placed on the ground.

All experiments described below were performed on a flat carpet (figure 8*a*) or artificial grass (figure 8*b*). In each trial, we turned on the power while holding the robot body such that its arms did not touch the carpet surface, after which we placed the robot on the ground. First, we performed preliminary experiments on the undamaged robot in order to hand-tune its parameters so that it would exhibit locomotion. Then, we performed experiments to investigate the robot's adaptability to physical damage. Parameter values determined from preliminary experiments are listed in table 2; these remained unchanged for all robot configurations. The noise terms $\xi_{\mathrm{yaw},i}$ and $\xi_{\mathrm{pitch},i}$ were varied every 0.25 s. The number of trials performed is shown in table 1. In a similar manner to the behavioural experiments, several markers were attached to the robot's body during the experiments to measure the locomotion velocity and the inter-arm coordination index (figure 7*d*).

**Table 2.** Parameter values used in the experiments.

parameter	value	unit
*c*	1.33	s^−1^
*α*_yaw_	0.175	—
*α*_pitch_	3.49 × 10^−2^	—
*β*_yaw_	5.24	—
*β*_pitch_	1.95	—
*γ*_yaw_	2.09	—
*γ*_pitch_	3.14	—
*κ_f_*	2.86	—
*κ_s_*	1.72	—
*θ_th_*	0.94	—
*A*	0.90	—

First, we destroyed the distal part of the arms during locomotion (figure 8*a* and electronic supplementary material, video S1, 3 : 30–5 : 26). The robot adapted to the damage to its arms within a few seconds and maintained its locomotion by coordinating some of the remaining arms.

Next, to compare the locomotion of brittle stars and that of the robot quantitatively, we performed experiments by setting the direction of motion and configuration of the robot such that they were nearly identical to those in the behavioural experiments shown in figure 1*d*. We set *ψ_x_* = 0 and *ψ_y_* = *π*/4 for the configurations A, B, C, F, and G, and *ψ_x_* = 0 and *ψ_y_* = −*π*/4 for the configurations D and E; we maintained these values during each trial. In this experiment, aluminium plates were used for the distal parts of the arms.

We found that the robot moved in a manner similar to a brittle star immediately after it was powered on (figure 8*b*,*c* and electronic supplementary material, video S1, 5 : 27–5 : 38 and video S4). This result was evaluated using the inter-arm coordination index *E_ij_* and the locomotion velocity for which the length scale was normalized with respect to the disc length. The results obtained for these indices are shown in figure 3. Both the *E_ij_* profiles and the locomotion velocity are generally in good agreement with those of brittle stars, although there are slight differences. This result indicates that the proposed control mechanism could be the mechanism underlying brittle star locomotion. Indeed, the proposed mechanism is in agreement with the biological finding that brittle stars can detect mechanical stimuli to their arms [34]. Further, we observed that the locomotion velocity of the robot does not decrease significantly except in configurations F and G, where only the arm oriented towards the direction of motion can strike the ground (figures 3*a* and 8*c*). Thus, the robot does not suffer from significant performance degradation even after the amputation of several arms.

Finally, in a similar manner to the behavioural experiments shown in figure 4*a*,*b*, the robot was placed on a flat stage so that its arms did not touch the ground. The arms did not coordinate but moved randomly (figure 4*c*,*d* and electronic supplementary material, video S5), which agrees with the behavioural experiments.

## 6. Discussion

We showed that our robot can adapt to physical damage within a few seconds without any contingency plan to anticipated failure modes; thus, it is considerably faster than conventional robots, whose adaptation time ranges from several tens of seconds to several minutes [9–16]. This drastic improvement was achieved by exploiting the inter-arm coordination mechanism of a brittle star, a primitive creature with expendable body parts.

We believe that the proposed control scheme is not limited to our brittle star-like robot in its application but has scope for more generic application, although there still exists a limitation. In fact, our control scheme can be interpreted from a broader design perspective: there are several distributed controllers, and the action generated by each is enhanced if the local sensory information is satisfactorily matched with an expectation and vice versa. An advantage of this design perspective is that it does not involve the solution of a complex optimization problem for the entire system but only requires a small amount of calculation at the local level. Hence, such a design is expected to enable robots to adapt to physical damage in real time and is applicable to unforeseen circumstances such as disaster scenarios.

**Figure 9.**
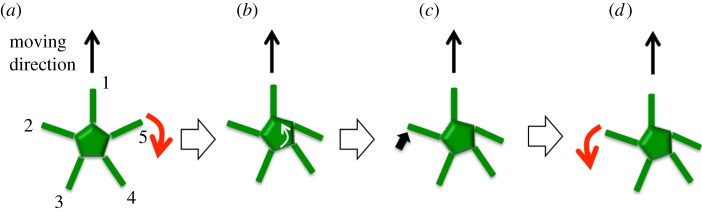
Qualitative explanation of the mechanism of inter-arm coordination. (*a*) Suppose that arm 1 is oriented towards the direction of motion (black arrow) and that arm 5 strikes the ground (red arrow). (*b*) The central disc rotates because of the counterbalance torque generated (white arrow). (*c*) Owing to the displacement of the proximal end of arm 2, the assistive reaction force acting on its tip increases (thick black arrow). (*d*) The local reflex works such that arm 2 strikes the ground (red arrow). This physical interaction works such that arms 2 and 5 strike the ground simultaneously.

Our findings have biological implications as well. This study shows that arm coordination emerges via physical interaction. In the case of subjects with five trimmed arms (figure 1*d*(A)), the reason why the two arms adjacent to the arm oriented towards the direction of motion tended to strike the ground periodically and synchronously is explained as follows (figure 9). When one of the arms strikes the ground (figure 9*a*), the central disc rotates because of the counterbalance torque generated (figure 9*b*). Then, owing to the displacement of the proximal end of the other arm, the assistive reaction force acting on its tip increases (figure 9*c*); hence, the local reflex works such that it strikes the ground (figure 9*d*). Consequently, the two arms tend to strike the ground simultaneously. From this consideration, we can conclude that physical interaction is likely essential for the inter-arm coordination in brittle stars, although neural control might play a certain role. While the importance of physical interaction is suggested in other works [29,30,35], this study indicates that physical interaction can be also exploited for the coordination of body parts that enables quick damage response.

It has been suggested that adaptive and resilient locomotion in animals in which multiple body parts are coordinated appropriately is generated by the close interaction between the nervous system, the body and the environment [29,30]. However, the essential mechanism of such locomotion remains unknown. The fundamental principles underlying adaptive and resilient locomotion can be determined on the basis of the decentralized control mechanism of brittle stars that has been described herein, and this may lead to a deeper understanding of inherent neuro-musculoskeletal functions in animals.

## Supplementary Material

Copy of supplementary material.xlsx

## Data accessibility

Data are available in the electronic supplementary material.

## Appendix A. Method for obtaining micro-computed tomography images

To obtain the X-ray micro-computed tomography (CT) images shown in figure 1*b* and electronic supplementary material, video S1, 1 : 13–1 : 31, the brittle star was fixed with Bouin's fluid and stained using 1% iodine metal (I2) dissolved in 100% ethanol [36]. Then, the brittle star was scanned using X-ray micro-CT (inspeXio SMX-100CT, Shimadzu Corporation, Kyoto, Japan), and the scanned images were saved in voxel format. The scanned images were then reconstructed three-dimensionally using VGStudio MAX (Volume Graphics, Heidelberg, Germany).
